# In silico analysis of a novel causative mutation in Cadherin23 gene identified in an Omani family with hearing loss

**DOI:** 10.1186/s43141-020-0021-4

**Published:** 2020-03-02

**Authors:** Mohammed Nasser Al-Kindi, Mazin Jawad Al-Khabouri, Khalsa Ahmad Al-Lamki, Flavia Palombo, Tommaso Pippucci, Giovanni Romeo, Nadia Mohammed Al-Wardy

**Affiliations:** 1 Department of Biochemistry, College of Medicine and Health Sciences, Sultan Qaboos University, P.O. Box 35, Al-Khoud, 123 Muscat, Oman; 2 Department of Otolaryngology and Head and Neck Surgery, Al Nahda Hospital, Ministry of Health, Muscat, Oman; 3 Medical Genetics Unit, Polyclinic Sant’Orsola-Malpighi, University of Bologna, Bologna, Italy

**Keywords:** CDH23, d2484a, In silico, Oman

## Abstract

**Background:**

Hereditary hearing loss is a heterogeneous group of complex disorders with an overall incidence of one in every 500 newborns presented as syndromic and non-syndromic forms. Cadherin-related 23 (CDH23) is one of the listed deafness causative genes. It is found to be expressed in the stereocilia of hair cells and in the retina photoreceptor cells. Defective CDH23 have been associated mostly with prelingual severe-to-profound sensorineural hearing loss (SNHL) in either syndromic (USH1D) or non-syndromic SNHL (DFNB12) deafness. The purpose of this study was to identify causative mutations in an Omani family diagnosed with severe-profound sensorineural hearing loss by whole exome sequencing technique and analyzing the detected variant in silico for pathogenicity using several in silico mutation prediction software.

**Results:**

A novel homozygous missense variant, c.A7436C (p. D2479A), in exon 53 of CDH23 was detected in the family while the control samples were all negative for the detected variant. In silico mutation prediction analysis showed the novel substituted D2479A to be deleterious and protein destabilizing mutation at a conserved site on CDH23 protein.

**Conclusion:**

In silico mutation prediction analysis might be used as a useful molecular diagnostic tool benefiting both genetic counseling and mutation verification. The aspartic acid 2479 alanine missense substitution might be the main disease-causing mutation that damages CDH23 function and could be used as a genetic hearing loss marker for this particular Omani family.

## Background

With the high rate of consanguineous marriages, several inherited diseases have been diagnosed among the Arab population including syndromic and non-syndromic deafness. A survey conducted in 2016 indicated that up to 49% of Omani marriages were consanguineous [1]. As a result, 70% of hearing loss cases in Oman were reported as possible inherited forms and until now, two genes have been reported to be involved in non-syndromic autosomal recessive genetic deafness in Oman, MYO15A, and Otoferlin [2–4].

There are no exact statistical figures of syndromic or non-syndromic hearing loss in Oman. However, worldwide studies revealed that approximately 466 million people (5.0%) of the world’s population were suffering from hearing loss [5]. Earlier studies highlighted that about 30% of the total deafness cases are syndromic [6]. Usher syndrome (USH) is one of the syndromic deafness forms with an estimated prevalence of 1 in 6000 to 1 in 10,000 representing about 6% of the total congenital deafness and approximately 50% of hereditary deaf–blind individuals. USH is a genetic disorder accompanied by a dual sensory impairment, sensorineural hearing loss, retinitis pigmentosa, and variable vestibular dysfunction. Clinically, it is categorized into three subclasses: USH1, USH2, and USH3. USH1 is the most severe form, characterized by congenital severe to profound deafness, vestibular dysfunction, and prepubertal onset of visual loss. It accounts for 33 to 44% of USH cases. USH2 impact has been ranked from moderate to severe hearing loss with no vestibular dysfunction. It affects 56 to 67% of all USH patients. USH3 is less severe and is characterized by progressive hearing and vestibular function loss. It is found in 1 to 6% in the general population. However, in the Finnish and Ashkenazi Jews populations, it rises to about 40% [7–10]. So far, 13 genes have been identified to be involved in USH development (https://sph.uth.edu/Retnet/sum-dis.htm). Among these genes is cadherin-related 23 (CDH23), causing Usher syndrome type 1D (USH1D) [11, 12]. Studies revealed that a defective CDH23 gene plays an important role in developing Usher syndrome (OMIM #601067) where it accounts for up to 32% of USH1 cases [13]. More than 350 associated mutations have been reported as either homozygous nonsense, frame-shift, splice-site, or missense mutations [14, 15]. Defective CDH23 was also detected in autosomal recessive non-syndromic hearing loss (OMIM #601386) (DFNB12) where more than 24 associated mutations have been reported as missense mutations [16–19]. In the cell membrane, CDH23 interacts with procadherin 15 (PCDH15) to create stereocilia organization and hair bundle formation which reflects its importance in normal inner-ear mechanotransduction [20].

Next generation sequencing (NGS) made a big leap in genome DNA sequencing. A whole exome and a gene panel can be rapidly sequenced, and the abnormality and specificity of the genome can be detected in a short period. However, the Sanger principle remains a useful technique for sequencing a short DNA fragment and for the confirmation of the NGS findings.

In this study, we genetically analyzed an Omani family diagnosed clinically with severe to profound hearing loss. Mutation detection was performed by Illumina HiSeq2000 platform (Illumina Inc., San Diego, CA, USA) NGS technique. DNA of an affected family member was sequenced to identify the family-specific mutated gene loci. The Sanger sequencing technique (ABI 3130 xl) was then applied for the whole family and control samples to confirm the NGS findings. A homozygous missense mutation in exon 53 of the cadherin-related 23 gene (CDH23) was detected in all affected members but was absent in the normal family members and controls. Subsequently, in silico genetic testing was used to verify the pathogenicity of the identified mutation.

## Methods

This study was conducted by the Department of Biochemistry, College of Medicine and Health Sciences, Sultan Qaboos University, and the Department of ENT, Al Nahdha Hospital, Ministry of Health, Oman, with collaboration from the Medical Genetics Unit, Polyclinic Sant’Orsola-Malpighi, Bologna, Italy.

Clinical examination: Four affected members, two males and two females, from one Omani family of consanguineous marriage (degree of parental relatedness, first cousins) were enrolled in this study.

Clinical history and audiological evaluation were done at the ENT department, Al Nahdha Hospital. Clinical examination was conducted using standard pure tone audiometry (PTA), optoacoustic and acoustic emittance tests. Blood samples from patients and their close relatives were collected in EDTA tubes. Samples from 130 male and female individuals without any hearing or visual disorders were used as normal controls.

DNA extraction and sequencing: Qiagen kit (Qiagen, Hilden, Germany) was used to extract the genomic DNA from peripheral blood of all collected samples. DNA from an affected member was analyzed using the Illumina HiSeq2000 platform (Illumina Inc., San Diego, CA, USA) NGS technique. The Sanger sequencing method (ABI PRISM Big-Dye terminator cycle sequencing premix kit (PE Applied Biosystems, Austin, TX, USA) was used to sequence 442 base pairs including the variant site to confirm the NGS finding. The rest of the family members and 130 controls were also tested for the detected variant. Polymerase chain reaction (PCR) forward (5′TCAGTGTCAAATCTCCAGAG3′) and reverse (5′TTGGCAAAGATTTCTCCCAG3′) primers were designed to amplify and confirm the NGS detected variant.

In silico analysis: In order to evaluate the putative pathological nature of the detected missense variant, physical properties of the amino acids such as change in hydrophobicity, and the impact of the substitution on protein structure and function, were analyzed using the online software SIFT (http://sift.jcvi.org/www/SIFT_enst_submit.html) [21], Mutation Taster (http://www.mutationtaster.org) [22], Mutation Assessor (http://mutationassessor.org/r3/) [23], PolyPhen-2 (http://genetics.bwh.harvard.edu/pph2/) [24], Mutpred2 (http://mutpred2.mutdb.org/index.html) [25], and SNAP2 (https://rostlab.org/services/snap2web/) [26]. Additional software such as MUpro (http://mupro.proteomics.ics.uci.edu/) [27], I-Mutant 2.0 (http://folding.biofold.org/i-mutant/i-mutant2.0.html) [28], and DUET (http://biosig.unimelb.edu.au/duet/) [29] were used to predict the impact of the mutation on protein structure stability based on the change in Gibbs free energy (ΔΔG).

The protein secondary structure was visualized and annotated using the online POLYVIEW (http://polyview.cchmc.org/) [30]. RMSD (root-mean-square deviation) and the template modeling TM-score (CαG alignment) (https://zhanglab.ccmb.med.umich.edu/TM-score/tmp/339254.html) were used to calculate the atomic deviation and the degree of similarity by superimposing models of native and mutant proteins [31, 32]. The variant genetics database including its location verification on the whole genome, cDNA, and protein sequences were retrieved by using the NCBI (National Center for Biological Information) (http://www.ncbi.nlm.nih.gov) and Ensemble genome browser (https://asia.ensembl.org/) online programs [33]. PrimerZ (http://genepipe.ncgm.sinica.edu.tw/ primerz/beginDesign.do) and BioEdit (http://www.mbio. ncsu.edu/ BioEdit/bioedit.html) were used for DNA primer design and sequence alignment [34, 35]. Validation of the protein degree of activity and function, the residues percentage of distribution of three regions, favored, allowed and residue in outlier region were analyzed and calculated using Ramachandran plot and the online software RAMPAGE (http://mordred.bioc.cam.ac.uk/ ~rapper/rampage.php) [36]. Attribution of the residue position to the protein function was analyzed by SWISS-MODEL template library (http://swissmodel.expasy. org) and visualized by Swiss-PDBViewer [37], Chimera program (https://www.cgl.ucsf.edu/chimera/download.html) [38], and Jmol (http://jmol.sourceforge.net) [39]. The retrieved variant was subjected to further analysis to predict the rate of conservation by alignment with close living species by using the ConSurf Server analysis package (http://consurf.tau.ac.il/2016/) [40], NCBI multiple sequence alignment (MSA) (https://www.ebi.ac.uk/Tools/msa/muscle/) [41], and Crustal Omega (http://www.ebi.ac.uk/Tools/msa/clustalo/) [42].

## Results

### DNA sequencing

**Fig. 1:**
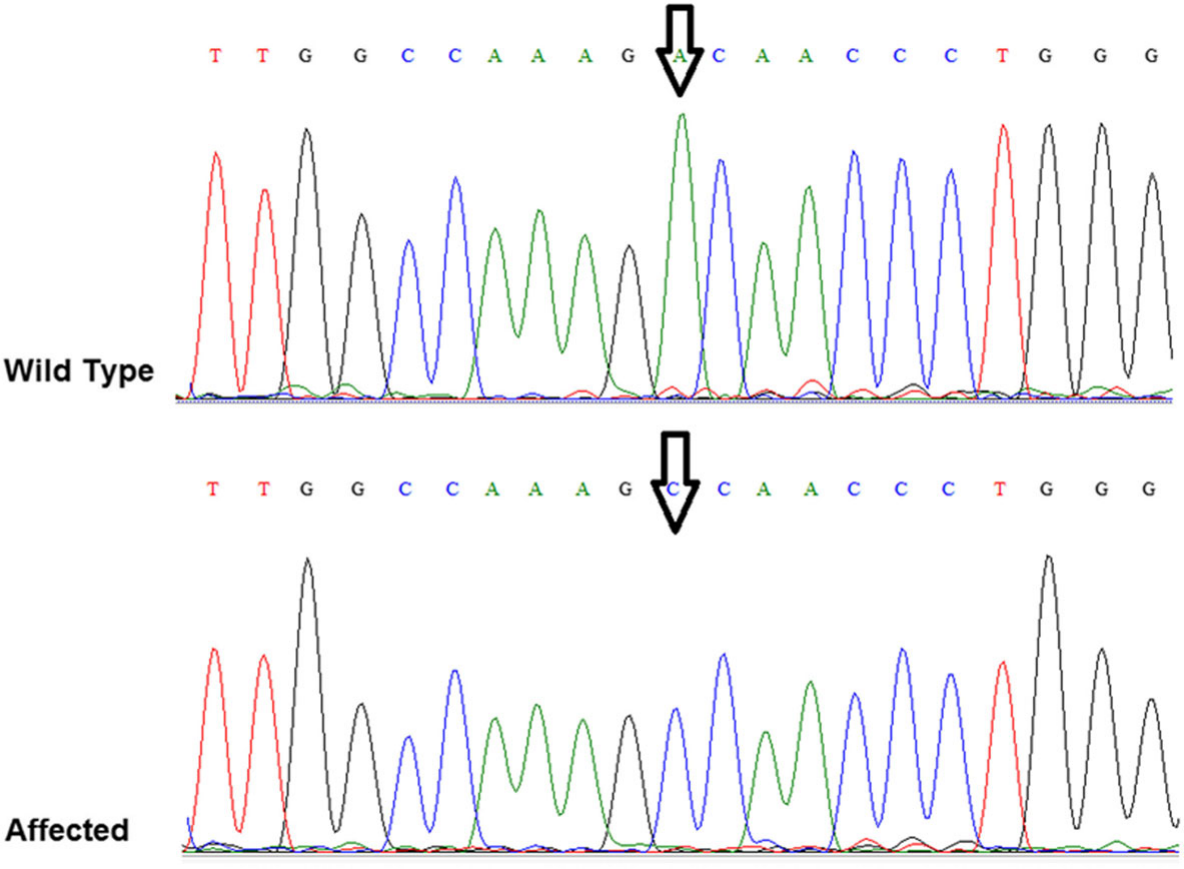
Electropherogram of CDH23 gene mutation position. Wild-type sequence represents normal control sample with AA genotype. Affected represents a deaf family member with CC genotype

The genetic abnormality of the affected family members diagnosed with hearing loss was detected by next generation sequencing whole exome technology. A novel homozygous missense variant, g.A71800709C, c.A7436 C, replacing the negatively charged aspartic acid residue with a nonpolar aliphatic amino acid alanine at position D 2479A in exon 53 of CDH23 gene was confirmed and verified by Sanger sequencing (Fig. 1).

**Fig. 2:**
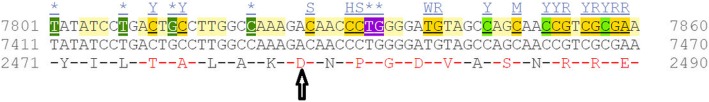
Normal cDNA of CDH23 transcript containing GAC (aspartic acid)

The CDH23 gene located on chromosome 10 contains 70 exons as illustrated on ensemble protein transcript CDH23 ENST00000224721.12, ENSG00000107736, Pfam: PF00028, and UniProtKBA0A0A0MQS6. Figure 2 illustrates the normal cDNA of CDH23 transcript as obtained from Ensemble genomic browser.

The CDH23 transcript (A0A0A0MQS6) was selected for further analysis to identify functional protein domains using the online SMART program. The program detected one signal peptide, 26 cadherin repeats (CA), also known as extracellular cadherin (EC) domains, one transmembrane region, and one low complexity region. The variant was found on domain 23 of the 26 CA (Fig. 2).

### In silico mutation analysis

The detected missense point variant was evaluated for its pathogenicity using different mutation prediction programs (Table 1) and was considered to be deleterious and damaging.

**Table 1:** Online in silico analysis tools used for CDH23 mutation pathogenicity prediction

Tool	Prediction value
SIFT	0.00
Polyphen	1.000
Provean	− 5.48
MutPred2	0.854
SNAP2	19
Mutation Assessor	FI score 4.485
Mutation Taster	Causing disease (0.99)

A variant is considered to be deleterious and damaging if the predicted values are as follows: SIFT > 0.05 (damaging), Polyphen < 0.8 (probably damaging), Provean > − 2.5 (deleterious), MutPred2 < 0.5 (deleterious), SNAPs < 0.00 non-neutral (deleterious), Mutation Assessor < 3.5 (high-functional, FI score 3.50–5.50), Mutation Taster < 0.5 (disease-causing)

The impact of the variant on protein stability changes was studied to evaluate its leverage on protein folding. The unfolding Gibbs free energy change (DDG or ΔΔG) was calculated using MUpro, I-Mutant 2.0, and DUET online tools. Models of native and mutant proteins were superimposed to predict the level of similarity between the two protein structures using the template modeling score (TM-score) and the root-mean-square deviation (RMSD) online software (Table 2).

**Table 2:** Online in silico analysis tools used for CDH23 protein stability changes and protein folding similarities

Tool	Prediction value
MUpro	DDG = − 0.58
I-Mutant 2.0 (kcal/mol)	DDG = − 1.22
DUET (kcal/mol)	DDG = − 2.88
TM-score	0.99786
RMSD (Å)	0.44

MUpro, I-Mutant 2.0, and DUET: DDG increases in protein stability > 0 > decrease in stability. 0.5 < TM-score < 1.00 reflects that the status of protein folding is identical. RMSD 0 Å − 2 Å reflects perfect match alignment

**Fig. 3:**
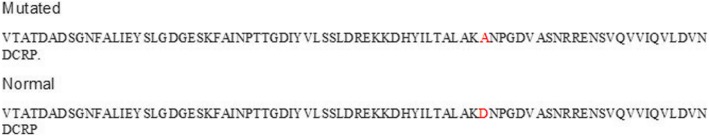
Mutated and normal CA-domain 23 amino acid sequence of CDH23. D2484A substitution is shown in the mutated (A) and normal (D) domain

The domain of interest was further analyzed for secondary structure prediction. Polyview-2D was used to predict the possible effects of the detected variant on the confirmation of domain 23. The impact of amino acid exchange on domain structure was evaluated by comparing wild-type predicted secondary structures and mutant sequences. The mutated domain structure was predicted to consist of 292 coils, 218 strands, and 28 helices compared to 302 coils, 215 strands, and 21 helices in the wild type (Fig. 3).

**Fig. 4:**
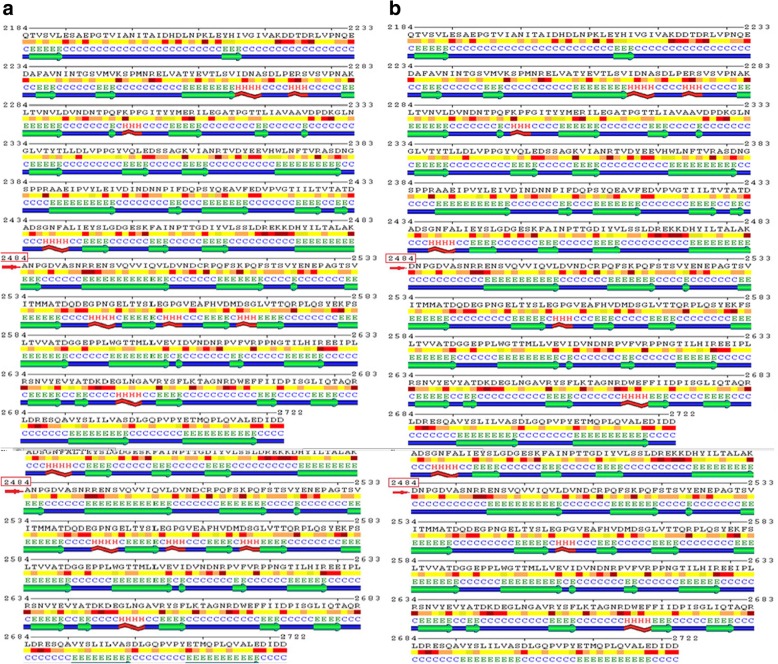
Secondary structure of CDH23 model-template alignment (5szn.1.A). **a** 2484 ALA predicted within beta strand. **b** 2484 ASP predicted within coiled coil. The red color indicates the highest level of amino acid conservation, while yellow indicates the lowest

**Fig. 5:**
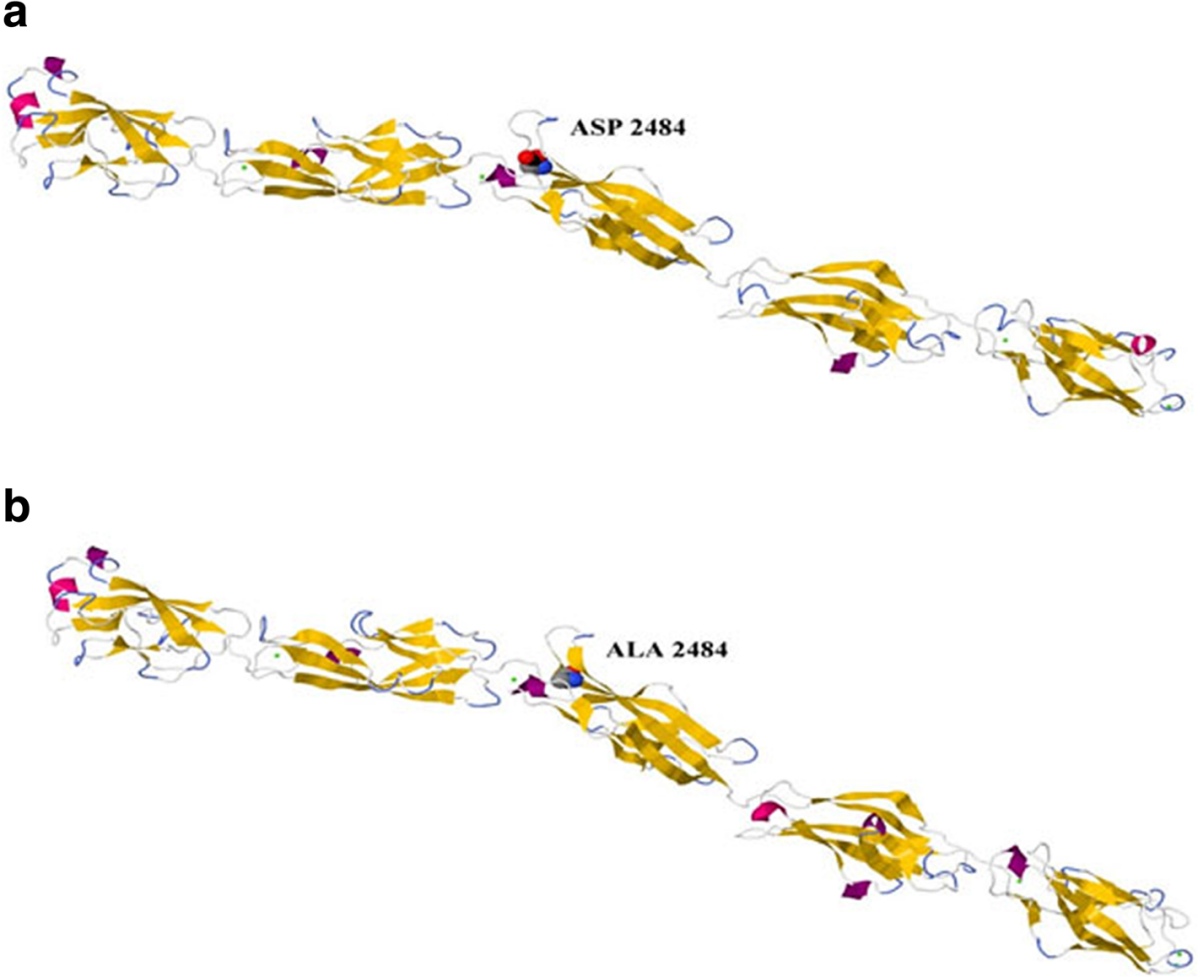
Molecular modeling of wild-type and mutant CDH23. Location of **a** the wild-type ASP (aspartic acid) as a part of the B strand and **b** the mutated ALA (Alanine) as a part of the turning loop of the extracellular cadherin (EC) domain 23 of CDH23 model

**Fig. 6:**
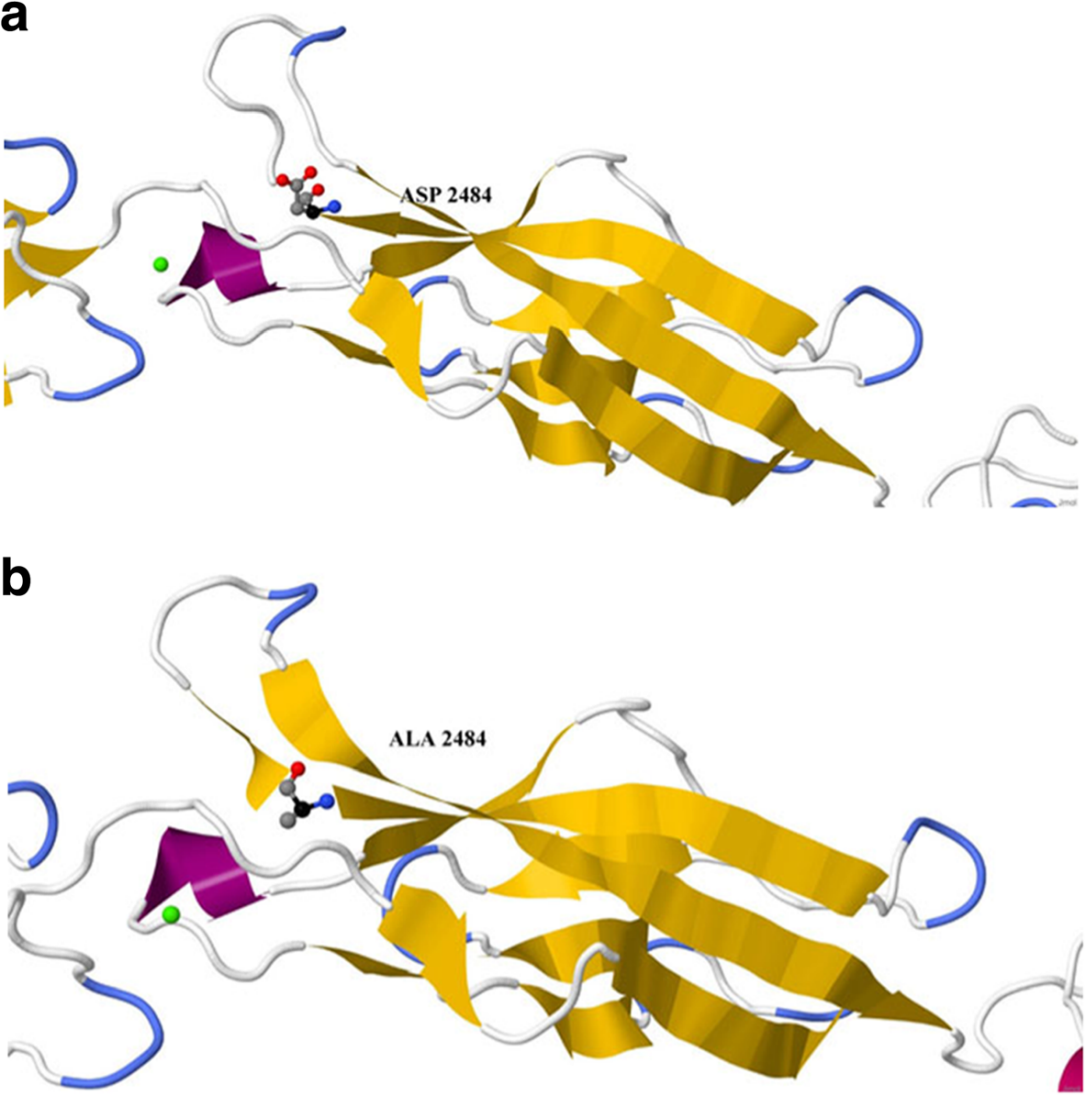
Magnified EC23 and the location of **a** ASP and **b** ALA of CDH23 model. Calcium molecules (red balls) are clearly seen as part of the structure

**Fig. 7:**
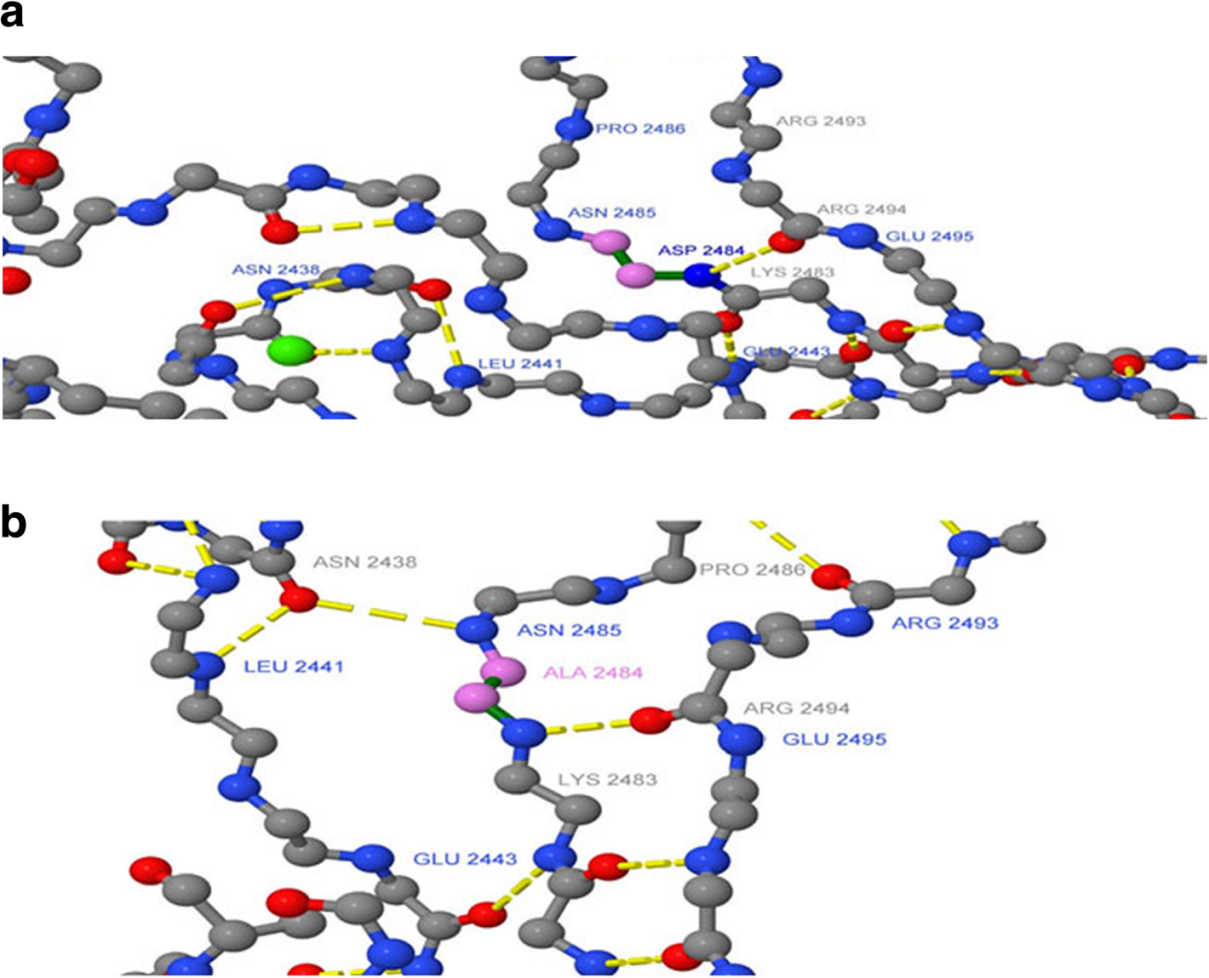
Predicted crystal native and mutant protein secondary structures. Interaction of CA 23 amino acids to form the CDH23 secondary structure. **a** The position of the native ASP 2484 and **b** the mutated ALA 2484. Hydrogen bonds are indicated with yellow lines

CDH23 transcript (A0A0A0MQS6) for the wild and mutated types was analyzed by the Swiss model program to build up possible protein templates and models. 5szn.1.A was selected to be the template and building model for CDH23 because of its similar identity with the wild and mutated types (33.96 and 34.52%, respectively). The D2479A ensemble position moved to D2484A on the 5szn model. Jmol package and Ramachandran plots were used to align and validate the two 3D structures in order to predict the possible impact of the mutated amino acid on CDH23 protein structure (Figs. 4, 5, 6, and 7).

**Fig. 8:**
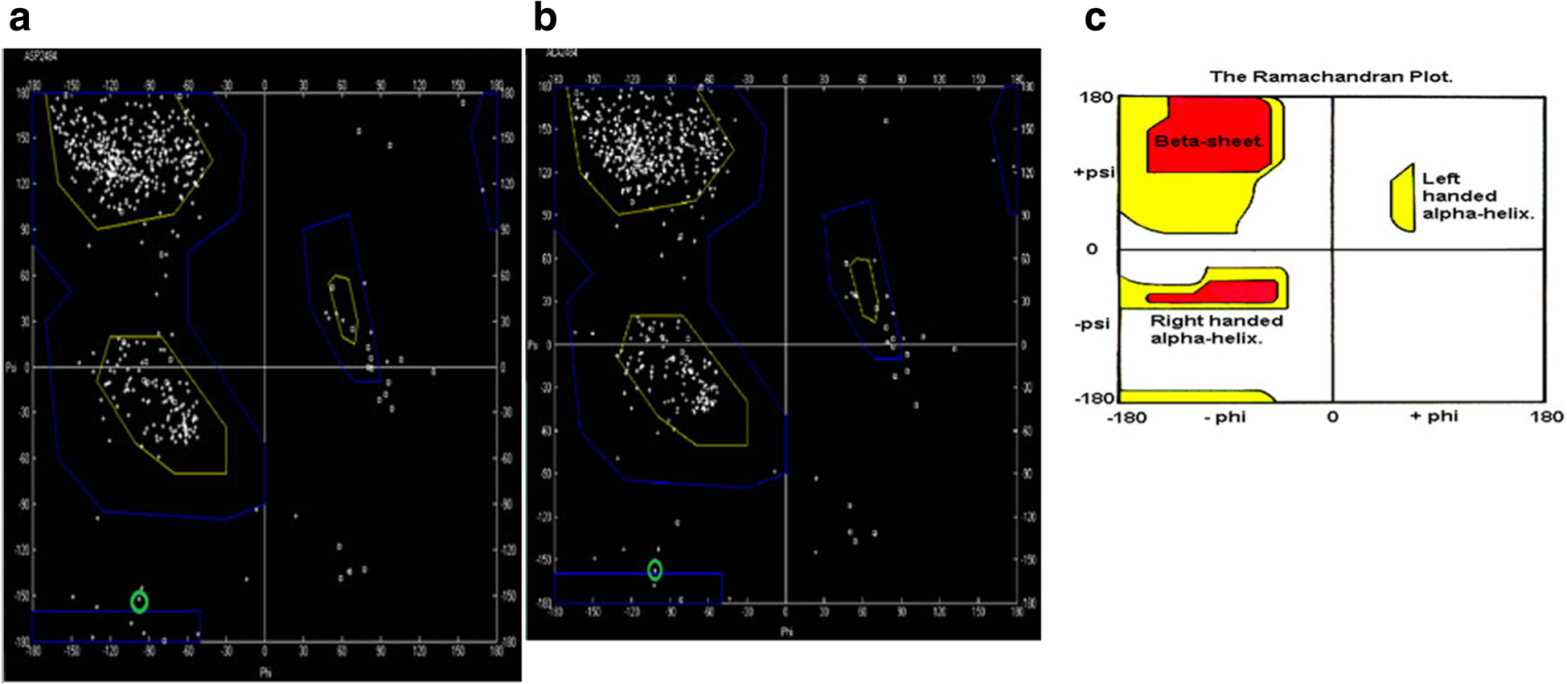
The Ramachandran plot for the CDH23 2484 residue. **a** Native CDH23 with ASP at position 2848. **b** Mutant CDH23 with ALA at position 2484. **c** Normal distribution of Ramachandran plot

The Ramachandran plot was used to calculate and visualize the dihedral angles predicting the energetically allowed residues based upon their phi and psi dihedral angles. A score of ≥ 90% in the allowed regions shows that the built model has high quality (Fig. 8 and Table 3).

**Table 3:** RAMPAGE: assessment of the Ramachandran plot

Model	Residues in favored regions		Residues in allowed regions		Residue in outlier region	
	No. of residues	% of residues	No. of residues	% of residues	No. of residues	% of residues
Wild-type residue [A2484:ASP] (− 97.36, − 152.49) in allowed region	505	94.0	25	4.7	7	1.3
Mutant-type residue [A2484:ALA] (− 101.70, − 157.59) in allowed region	506	94.2	24	4.5	7	1.3

**Fig. 9:**
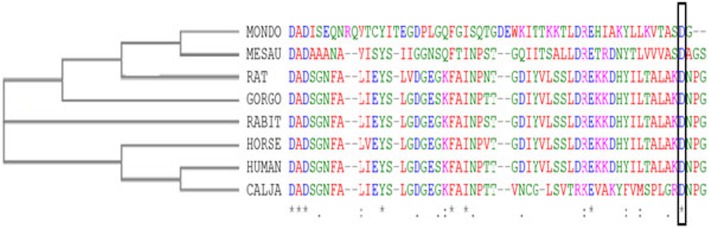
Conservation analysis of aspartic acid 2479 residue. CA 23 of Human CDH23 alignment with other mammalian protein sequences. Phylogenetic tree and MSA sequence alignment of ASP (the black block) degree of conservation of Human and other mammalian species

**Fig. 10:**
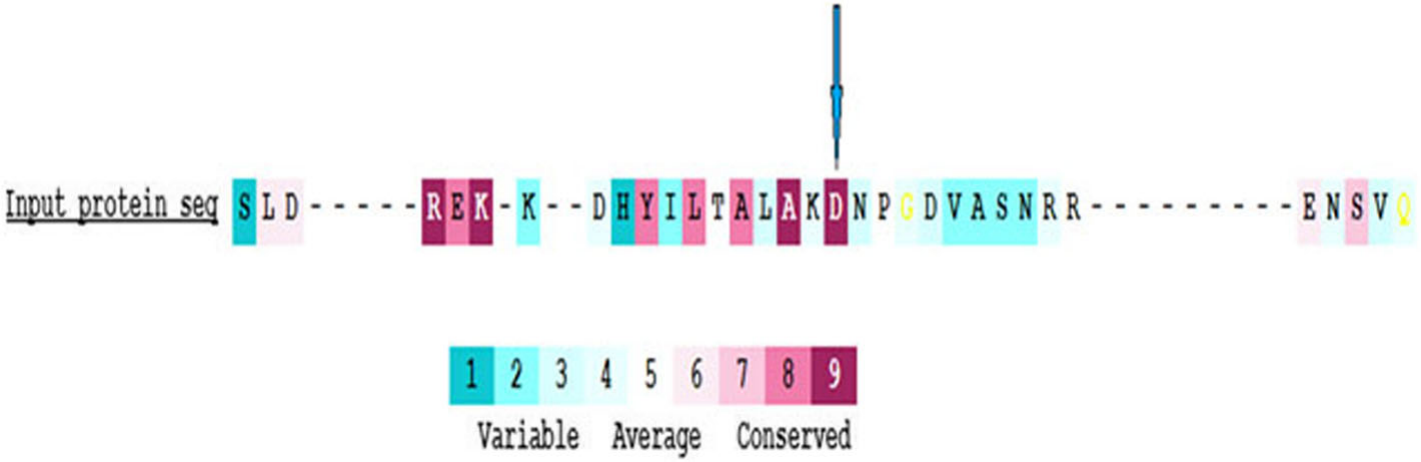
The position of ASP (D) predicted to be within the highest conserved region with a value of 9

The evolutionary conservation rate of the substitution was analyzed using the online NCBI protein cluster (Fig. 9) and ConSurf programs (Fig. 10).

## Discussion

CDH23 is an adhesive protein expressed in the neurosensory epithelium of the inner ear hair cells and encodes the transmembrane Ca^2+^-dependent adhesion protein, cadherin 23 (CDH23) [43]. It is thought to be involved in stereocilia organization and hair bundle formation [44]. Using its adhesion property, it interacts with protocadherin15 protein to form a tip-link filamentous complex, which is the main component that drives the normal mechano-transduction process in auditory and vestibular hair cells. Hence, a change in the protein structure might lead to a significant defect in its comprehended performance, which, in turn, could terminate the entire inner ear mechano-transduction process by turning off the sound perception and acceleration stages. The impact of the defective CDH23 protein was observed in both syndromic and non-syndromic hearing loss forms [11, 43]. It accounted for up to 32% of Usher syndrome type 1 cases [13]. More than 24 associated mutations have been reported as missense mutations that clearly appeared as an important cause of hearing loss in Asian populations [16–19]. Recent research studies suggest that in silico mutation prediction might be used as a first-line molecular diagnosis tool serving both genetic counseling and mutation verification and variant classification [45, 46]. Prediction of variant pathogenicity using bioinformatics tools was conducted by several studies. A homozygous c.5985C > A (p.Y1995X) variant, a heterozygous p.E1006K, and p.D1663V were detected in the Chinese population [47, 48]. The mutation frequency spectrum of CDH23 among the recessive inherited cases is 5.7% in the Japanese population and 15% in the Korean population [16, 19, 49]. Other gene variants were also analyzed using such programs such as V66 M variant of human BDNF in psychiatric disorders and computational modeling of complete HOXB13 protein for predicting the functional effect of SNPs and the associated role in hereditary prostate cancer [50, 51]. The American College of Medical Genetics and Genomics (ACMG) guide for the interpretation of sequence variants elaborated the usefulness of the predictive software programs for risk estimation and accurate interpretation of the potential causality of sequence variation [52]. The variant specifications (location), classification (mutation type), and pathogenicity degree interpretation (pathogenic, likely pathogenic, uncertain significance, likely benign, and benign) were thoroughly revised by the ACMG, and the use of specific standard terminology in describing the variant identity was recommended [45, 46].

In this study, we genetically analyzed an Omani family who was diagnosed clinically with severe to profound hearing loss. The analysis revealed a missense variant on CDH23 (c.A7436C) which was detected by NGS technology and confirmed by Sanger DNA sequencing methodology. Affected family members had the CC genotype, while all 120 normal control samples had the wild-type genotype of AA. The identified variant was subjected to various in silico functional prediction algorithm software to evaluate the pathogenicity level, functionality, and protein stability. The evaluation was based on criteria such as variant location on the genome, sequence homology, conservation level, and physicochemical and structural properties. Sorting Intolerant from Tolerant (SIFT), Polymorphism Phenotyping 2 (PolyPhen 2), PROVEAN (Protein Variation Effect Analyzer), MutPred2 (Mutation Prediction), screening for non-acceptable polymorphisms (SNAPs), and Mutation Assessor protein function prediction software were used, and all agreed on the pathogenicity of the variant. The results from these predictors classified the variant as damaging, deleterious, and disease-causing, and boosted the variant damaging level on the mutant protein structure.

Prediction of the variant’s influence on the stability of protein structure is a crucial aspect for studying the function of the protein. The unfolding Gibbs free energy change (ΔG) of the native and mutant structures was calculated by subtracting the free energy change of the mutant protein from the free energy change of the native protein (Kcal/mol) (DDG or ΔΔG)  = ΔG mutant – ΔG wild type. Above zero value of DDG predicts high stability of the mutant protein and a score below zero predicts low stability [53, 54]. Structure stability was predicted by using I-Mutant 2.0, MUpro, and DUET programs. All analyses agreed that variant p.D2479A might destabilize the protein structure by indicating a negative score.

Alignment and proteins similarity are important factors assessing generated protein models of related identity. The template modeling score (Tm-score) was used to determine the topological deviation of native from mutant model structures, whereas RMSD was used to calculate the average distance of the alpha carbon backbones between the two models [55]. Both programs predicted a perfect match between the two model structures—wild type and mutant. The sequence of amino acids determines the protein conformation, and the physical and chemical properties of the amino acids greatly affects protein function. Alanine, known as a strong helix-favoring residue, engages in van der Waals interactions, nonpolar and uncharged (hydrophobic) status. Aspartic acid, on the other hand, is negative in charge, polar, and able to make hydrogen bonds with other amino acids and water (hydrophilic status). The substitution in this case might change the protein self-interaction. Therefore, secondary and tertiary protein structures were further analyzed to investigate the impact of the mutant variant on the protein function. According to the Polyview-2D results, the mutated domain structure was predicted to consist of 292 coils, 218 strands, and 28 helices compared to 302 coils, 215 strands, and 21 helixes in the wild type. It is clearly seen that alanine is located within the β-strand segment of the mutated protein whereas aspartic acid is located within the coiled loop of the wild-type protein [56, 57]. Templates and models for both proteins were created by using the Swiss model online program. The complete protein structure of the CDH23 protein was not available in the Protein Data Bank (PDB). Hence, PDB files for both mutant and native proteins were modeled by Swiss model online program. The two models supported the Polyview-2D findings. PDB files were analyzed and visualized by SWISS PDB Viewer and Jmol. The difference in hydrogen bonding on the native and mutant protein domain was calculated. Both native and mutant variants were hydrogen-bonded with ARG 2494. However, asparagine at position 2485 bonded with asparagine at position 2438 in the mutated but not in the wild-type form. This could indicate that changing the amino acid aspartate to alanine could affect protein structure and, therefore, its function. CDH23 gene information was retrieved from NCBI, Ensemble genome browser, and UniProt database. Analysis showed that the detected variant is a novel homozygous missense variant located at g. 71800709, c.A7436C, p.D2479A, chr10:71800709, q22.1 in exon 53, of ENST00000224721 of GRCh38.p7. The exact location of variant domain on the protein was predicted using Smart genetics program and was found to be within the CA-domain 23. The domain consists of 84 amino acids ordered from 2424 to 2507 and is reported as cadherin repeat in the extracellular domain of a transmembrane CDH23 [58]. The total number of repeats is 27 presented within the adherent junctions region as a glycoprotein. The EC domains are involved in cell-to-cell adhesion via hemophilic calcium-dependent interactions [59]. Binding of calcium to the EC domains at the linker region between consecutive EC repeats promotes the linearization, rigidity, and dimerization of CDH23 [60]. The aspartic acid residues have a high Ca^+^ affinity, and that may play an important role in the interactions of CDH23 molecules either with CDH23 or with other proteins. Since calcium provides rigidity to the elongated structure of cadherin molecules and enables hemophilic lateral interaction, the mutation is likely to result in a decreased affinity for calcium and, in turn, impairs the whole process of protein interaction [61]. A Phi/Psi two torsion angles N-Cα (called Phi, φ) and Cα-C (called Psi, ψ) in a polypeptide chain play a role in the control of local structure folding. Therefore, applying Ramachandran plot would predict the protein folding capability and, in turn, predict the quality of the three-dimensional structures. A Ramachandran plot was obtained to validate the protein structures that were created by the Swiss model for both mutant and native template models. Swiss PDB viewer was used to create Ramachandran plot, and Rampage program was used to calculate the amino acid assembly point percentage. According to the program, a good protein structure model is expected to have more than 90% of the residues within the core or favored region of the protein. RAMPAGE predicted greater than 94% of the 537 residues assembled within the favored region of both native and mutant proteins [62]. Conserved amino acids in proteins are found to be involved in various cellular processes in a biological system including genome stability [63]. Due to this, phylogeny and multi-sequencing alignment (MSA) were conducted to evaluate the aspartic acid 2484 stability and conservation status. As was predicted by ConSurf Server package and Polyview-2D, aspartic acid is highly conserved with a score of 9 among species reflecting the importance of this amino acid position that may play a crucial role in the integrity of protein structure and conformation.

One limitation of this study is that the detected mutation was identified by next generation sequencing technology, which requires sequencing of the whole human exon. The technique is outside the routine daily assays, and the running cost is high. However, the mutation confirmation assay by Sanger DNA sequencing technology is more economical.

## Conclusion

In this study, we used various in silico mutation prediction programs to analyze a substituted variant on CDH23 protein. The variant was typed on an Omani family diagnosed clinically with hearing loss. The analysis predicted the novel substituted D2479A to be deleterious and protein destabilizing mutation at a conserved site on CDH23 protein. This mutation might lead to a major disruption in CDH23 protein structure that may cause disturbance of stereocilia organization and hair bundle formation affecting the mechano-transduction process and, in turn, hearing loss. In silico mutation prediction analysis might be used as a useful molecular diagnostics tool benefiting both genetic counseling and mutation verification in the governmental and private sectors. The affected family might benefit from the outcome of this research by considering the potential risk of consanguineous marriage.

## Data Availability

All data generated or analyzed during this study are included in this published article.

## Abbreviations

ALA: Alanine
ASP: Aspartic acid
CDH23: Cadherin-related 23
DDG or ΔΔG: Gibbs free energy change
EC: Extracellular
MSA: Multiple sequence alignment
MutPred2: Mutation prediction
NCBI: National Center for Biological Information
NGS: Next generation sequencing
PCDH15: Procadherin 15
PolyPhen 2: Polymorphism phenotyping 2
PROVEAN: Protein Variation Effect Analyzer
PTA: Pure tone audiometry
RMSD: Root-mean-square deviation
SIFT: Sorting Intolerant from Tolerant
SNAPs: Screening for non-acceptable polymorphisms
SNHL: Sensorineural hearing loss
TM-score: Template modeling score
USH: Usher syndrome
